# Thalline, a New Antipyretic

**Published:** 1886-05

**Authors:** 


### THALLINE, A NEW ANTIPYRETIC.

Pavay has for some months been studying the properties of *thalline*, and has made a large number of clinical observations. The following are his conclusions:

1. Thalline is a powerful antipyretic agent, even in small doses. From 25 to 75 centigrammes (4 to 12 grs.) will reduce the bodily temperature from 1 to 4 degrees centigrade.

2. The reduction of the temperature takes place within from one-half hour to two hours after the administration of the medicine. The effect endures from two to four hours, rarely for eight hours. The temperature then rises rapidly, being usually preceded by chilly sensation, sometimes by a true rigor.

3. When the effect of the thalline is exhausted, a fever more intense than before the administration of the drug is observed.

4. Thalline exerts no influence upon the pulse or respiratory movements.

5. After the administration of thalline, the patient perspires very freely.

6. Thalline produces neither malaria, vomiting, deafness, epigastric pain, epistaxis, nor tinnitus; but depression and cyanosis are sometimes observed.

7. When thalline is given hypodermically, its action is more rapid and enduring than when administered by the mouth.

8. Thalline much resembles kairine in its action, but acts more rapidly, powerfully and surely. Moreover, its secondary effects are not so injurious as those of kairine.

9. Thalline cannot be placed in the same rank as quinine, antipyrine, and salicylic acid.

10. Neither the duration nor course of infectious diseases are influenced by thalline.

11. All acute diseases, in which a rapid reduction of temperature is a desideratum, may be treated by thalline. The subcutaneous method of administration is to be preferred.

12. If the system is enfeebled, this drug must be given in small doses and with great caution; from 20 to 25 centigrammes (3 to 4 grs.) are sufficient.

13. The two salts of thalline (sulphate and muriate) have the same action.

14. Atropin will not arrest the perspiration produced by thalline.

15. The red blood corpuscles are not destroyed by thalline.

According to the author, the rapid and powerful effects of thalline can be explained only by assuming that it acts upon the nervous centers which control the temperature of the body.—*Bull. Gen. de Therapeutique.*

